# A “love match” score to compare root exudate attraction and feeding of the plant growth-promoting rhizobacteria *Bacillus subtilis*, *Pseudomonas fluorescens*, and *Azospirillum brasilense*

**DOI:** 10.3389/fmicb.2024.1473099

**Published:** 2024-09-23

**Authors:** Eulalie Fourneau, Mélissa Pannier, Wassila Riah, Emmanuelle Personeni, Annette Morvan-Bertrand, Josselin Bodilis, Barbara Pawlak

**Affiliations:** ^1^Univ Rouen Normandie, Normandie Univ, GLYCOMEV UR 4358, SFR Normandie Végétal FED 4277, Rouen, France; ^2^UniLaSalle Rouen, UR AGHYLE, UP2018.C101, SFR Normandie Végétal FED 4277, Mont-Saint-Aignan, France; ^3^Univ Caen Normandie, Normandie Univ, INRAE, UMR 950 EVA, SFR Normandie Végétal FED 4277, Caen, France

**Keywords:** plant growth-promoting rhizobacteria (PGPR), root exudates, rhizosphere microbiota, chemotaxis, bacterial growth, *Bacillus subtilis*, *Pseudomonas fluorescens*, *Azospirillum brasilense*

## Abstract

**Introduction:**

The rhizosphere is the zone of soil surrounding plant roots that is directly influenced by root exudates released by the plant, which select soil microorganisms. The resulting rhizosphere microbiota plays a key role in plant health and development by enhancing its nutrition or immune response and protecting it from biotic or abiotic stresses. In particular, plant growth-promoting rhizobacteria (PGPR) are beneficial members of this microbiota that represent a great hope for agroecology, since they could be used as bioinoculants for sustainable crop production. Therefore, it is necessary to decipher the molecular dialog between roots and PGPR in order to promote the establishment of bioinoculants in the rhizosphere, which is required for their beneficial functions.

**Methods:**

Here, the ability of root exudates from rapeseed (*Brassica napus*), pea (*Pisum sativum*), and ryegrass (*Lolium perenne*) to attract and feed three PGPR (*Bacillus subtilis*, *Pseudomonas fluorescens*, and *Azospirillum brasilense*) was measured and compared, as these responses are directly involved in the establishment of the rhizosphere microbiota.

**Results:**

Our results showed that root exudates differentially attracted and fed the three PGPR. For all beneficial bacteria, rapeseed exudates were the most attractive and induced the fastest growth, while pea exudates allowed the highest biomass production. The performance of ryegrass exudates was generally lower, and variable responses were observed between bacteria. In addition, *P. fluorescens* and *A. brasilense* appeared to respond more efficiently to root exudates than *B. subtilis*. Finally, we proposed to evaluate the compatibility of each plant–PGPR couple by assigning them a “love match” score, which reflects the ability of root exudates to enhance bacterial rhizocompetence.

**Discussion:**

Taken together, our results provide new insights into the specific selection of PGPR by the plant through their root exudates and may help to select the most effective exudates to promote bioinoculant establishment in the rhizosphere.

## 1 Introduction

Climate change is affecting agroecosystems by increasing the frequency of extreme weather events like droughts, floods, or heatwaves, therefore leading to biotic and abiotic stresses for plants (George et al., 2024). Moreover, contemporary intensive farming systems use high levels of fertilizers, which have negative environmental and health consequences. In order to feed the growing human population, increased global crop production is needed (Foley et al., 2011; Oburger et al., 2022). These challenges require sustainable agroecosystems that could rely on alternative plant protection products such as biopesticides, bioprotectants, or biostimulants, which can be substances or microorganisms (Lankinen et al., 2024). In particular, plant growth-promoting rhizobacteria (PGPR) would be good candidates as microbial inoculants for sustainable crop production due to their many beneficial effects (Shah et al., 2021). The rhizosphere, i.e., the soil zone surrounding plant roots, is a complex ecological niche that is directly influenced by rhizodeposits released by the roots which select soil microorganisms through specific interactions and signaling (Jacoby et al., 2020; Tian et al., 2020). Thus, by recruiting specific microorganisms, the plant assembles its rhizosphere microbiota, which plays an essential role in plant growth, health, and protection against biotic or abiotic stresses (Philippot et al., 2013; Qu et al., 2020; Thoms et al., 2021; Dlamini et al., 2022; Santoyo, 2022).

In particular, PGPR are beneficial members of the rhizosphere microbiota that can promote plant growth directly by enhancing nutrient availability through nitrogen fixation, phosphate solubilization, potassium solubilization, and iron uptake, or by phytohormone regulation or production such as auxins, cytokinins, gibberellins, abscisic acid, and ethylene (Tsukanova et al., 2017; Oleńska et al., 2020). PGPR can also act as biocontrol agents through antibiotic and lytic enzyme production, activation of plant induced systemic resistance (ISR), and competition for space and nutrients in the rhizosphere (Pathania et al., 2020; Shah et al., 2021). Among the many beneficial rhizobacteria identified, *Bacillus subtilis*, *Pseudomonas fluorescens*, and *Azospirillum brasilense* are well-known PGPR that are already used and commercialized as biofertilizer products (Basu et al., 2021). Indeed, *B. subtilis* can improve nutrient availability (N and P), modify plant growth hormone homeostasis, reduce both drought and salt stress, form biofilms, elicit ISR, degrade *quorum sensing* signals of some phytopathogens, and produce antimicrobial compounds such as lipopeptides (e.g., surfactin), exoenzymes, and volatile organic compounds (Blake et al., 2021). *P. fluorescens* can solubilize phosphate, trigger ISR, and produce phytohormones, hydrogen cyanide, antibiotics, and siderophores which chelate iron, therefore making it bioavailable for the plant and preventing its acquisition by phytopathogens (Meliani et al., 2017; David et al., 2018). Finally, *A. brasilense* can fix atmospheric nitrogen in a non-symbiotic process, synthesize several phytohormones, and increase tolerance to abiotic stresses like salinity or drought through osmolyte accumulation in plant cells and biotic stresses through ISR (Fukami et al., 2018).

While the microbiota acts on the plant, its roots release rhizodeposits, such as mucilage and root-associated cap-derived cells (AC-DCs) also known as border cells, which together form the root extracellular trap (RET), and they also include root debris and soluble root exudates (Jones et al., 2009; Driouich et al., 2021). Indeed, plants produce up to 200,000 metabolites, including those released by roots and AC-DCs in the rhizosphere which are estimated to represent approximately 5–20% of total photosynthetically fixed carbon (Huang et al., 2020; Sugiyama, 2019; Ma et al., 2022). In particular, root exudates, which are greatly involved in plant–microorganism interactions, can be defined as a complex and dynamic mixture of numerous low molecular weight compounds that are relatively water soluble and capable of diffusing into the soil, including primary metabolites such as sugars, amino acids, and organic acids, as well as secondary metabolites such as flavonoids or glucosinolates (Badri and Vivanco, 2009; Sasse et al., 2018; Sugiyama, 2019; Vives-Peris et al., 2020; McLaughlin et al., 2023). The amount and composition of root exudates, which are challenging to characterize, can vary during plant growth according to various factors such as physical factors like temperature, light intensity, CO_2_, drought, or flood; chemical factors like soil nutrients or heavy metal stress; and biological factors like the plant’s genotype and age, or the presence of herbivores, microorganisms, and other plants through allelopathy (Vives-Peris et al., 2020; Ma et al., 2022). Moreover, the chosen collection method (i.e., soil or hydroponic plant culture, sampling medium and duration, and sterility) quantitatively and qualitatively affects root exudates, and no standard method for collecting and analyzing exudates has been established so far (Oburger and Jones, 2018; Calabrese et al., 2023; McLaughlin et al., 2023).

Despite the challenge of characterizing root exudates, they have been shown to be particularly involved in regulating the rhizosphere microbiota assembly and its evolution over time (Ma et al., 2022). On the one hand, root exudates provide a rich source of organic carbon for rhizosphere microorganisms (Lei et al., 2023). These exudates have been shown to attract symbiotic microorganisms such as arbuscular mycorrhizal fungi or rhizobia with strigolactones or flavonoids, respectively (Oldroyd, 2013; Sasse et al., 2018). Other beneficial microorganisms can be attracted, such as PGPR, although their precise attractant signals are more delicate to identify (Hassan et al., 2019). On the other hand, plant roots can secrete antimicrobial compounds like proteins or secondary metabolites, as well as *quorum sensing* interference molecules that inhibit bacterial communication (Haichar et al., 2014; Tian et al., 2020).

Hence, in order to colonize the rhizosphere, soil microorganisms must be able to survive and establish themselves in this competitive environment, meaning they must feature rhizocompetence traits such as motility, ability to utilize root exudates as nutrients, and biofilm formation (Kaur et al., 2017; Santoyo et al., 2021). Among these behaviors, chemotaxis toward the roots through their exudates, and then root exudates degradation for bacterial growth are the first responses involved in the rhizosphere microbiota assembly. Chemotaxis is defined as the ability of motile bacteria to migrate toward an attractant gradient or away from a repellent one. By modifying the balance between their runs and tumbles, bacteria can move toward environments that are favorable for growth and survival (Porter et al., 2011; Ping et al., 2013; Bi and Sourjik, 2018; Grognot and Taute, 2021). Most soil bacteria are motile and capable of chemotaxis (Scharf et al., 2016). By perceiving root exudates through chemoreceptors, rhizobacteria can move toward the root and accumulate, therefore initiating rhizosphere colonization (Allard-Massicotte et al., 2016; Feng et al., 2021). Next, some studies have shown that rhizobacteria are able to grow by degrading nutrients contained in root exudates that are bioavailable for their catabolism (Guyonnet et al., 2018; Zhalnina et al., 2018; Carreras et al., 2019; Dhungana et al., 2023).

The rhizocompetence traits described earlier are essential for applied bioinoculants such as PGPR to survive and compete with other microorganisms in the rhizosphere (Kaur et al., 2017). Indeed, bioinoculants may prove to be unsuccessful under field conditions compared to their performance in laboratory assays (Thilakarathna and Raizada, 2017; O’Callaghan et al., 2022; Salomon et al., 2022). This challenge requires a better understanding of the mechanisms involved in the rhizosphere colonization by microorganisms and their selection by the plant through root exudates, in order to improve bioinoculant formulation and establishment (Fasusi et al., 2021; Kaur and Vishnu, 2022; O’Callaghan et al., 2022; Hossain et al., 2023; Poppeliers et al., 2023).

In order to gain a better understanding of the relationships between PGPR and their host plants, we sought to identify specific bacterial responses depending on the root exudates of each plant species. Therefore, we studied the behavior of three PGPR strains, *B. subtilis* ATCC 6633*, P. fluorescens* ATCC 17400, and *A. brasilense* Sp245, in response to root exudates from rapeseed (*Brassica napus*), pea (*Pisum sativum*), and ryegrass (*Lolium perenne*). These three plants were selected because they belong to different families (Brassicaceae, Fabaceae, and Poaceae) of agricultural interest, while the bacterial species are well-known PGPR commercialized as biofertilizers, and the chosen strains have been reported to exhibit plant growth-promoting properties (Gaballa et al., 1997; Bartolini et al., 2017; Méndez-Gómez et al., 2020; Planchon et al., 2021; Yadav et al., 2021; Yu et al., 2021; Zou et al., 2024). The ability of each plant’s exudates to attract and feed beneficial bacteria was measured and compared, and different responses were observed. Rapeseed exudates were the most attractive and induced the fastest growth, pea exudates allowed the highest biomass production, while ryegrass exudates were less efficient. Moreover, *P. fluorescens* and *A. brasilense* appeared to respond better to root exudates than *B. subtilis*. Finally, we proposed an evaluation of the performance of each plant–PGPR pair by assigning them a “love match” score. This scoring system may help to select efficient root exudates or co-cultivated plants that could be used in combination with a specific PGPR to enhance its rhizocompetence and thus its establishment in the rhizosphere microbiota.

## 2 Materials and methods

### 2.1 Bacterial strains and growth conditions

Bacterial strains *Bacillus subtilis* ATCC 6633, *Pseudomonas fluorescens* ATCC 17400, and *Azospirillum brasilense* Sp245 were grown on LB medium (10 g/L tryptone, 5 g/L yeast extract, and 5 g/L NaCl) or on M9 minimal medium (3 g/L KH_2_PO_4_, 0.5 g/L NaCl, 6.78 g/L Na_2_HPO_4_, 1 g/L NH_4_Cl, 2 mM MgSO_4_, 0.1 mM CaCl_2_, and 20 mM glucose) with stirring (120–140 rpm). For *A. brasilense*, M9 medium was modified from MMAB medium (Vanstockem et al., 1987) by replacing glucose with 2.5 g/L malate and adding 5 mg/L biotin and 2.5 mg/L FeSO_4_. Carbon concentrations of all three media were measured by isotope ratio mass spectrometry (IRMS) using the PLATIN’ platform (University of Caen Normandy). *B. subtilis* and *P. fluorescens* were grown at 30°C and *A. brasilense* at 25°C.

### 2.2 Plant material and growth conditions

Pea (*Pisum sativum* var. Astronaute) seeds were surface sterilized and sown on agar 1% as described by Calabrese et al. (2023). Germinated seeds were transferred to a hydroponic system with ¼ Hoagland nutrient solution (2.5 mM Ca(NO_3_)_2_•4H_2_O, 1.25 mM KNO_3_, 0.5 mM MgSO_4_, 0.25 mM KH_2_PO_4_, 0.2 mM Fe-Na EDTA, 14 μM H_3_BO_3_, 5 μM MnSO_4_, 3 μM ZnSO_4_, 0.7 μM (NH_4_)_6_Mo_7_O_24_, 0.7 μM CuSO_4_, 0.1 μM CoCl_2_) in a phytotronic chamber (day/night cycle: 16 h, 23°C/8 h, 20°C) up to the 6–8 leaf stage. Rapeseed (*Brassica napus* var. Aviso) seeds were sown in perlite and were then transferred to a hydroponic system with ¼ Hoagland nutrient solution in a greenhouse (day/night cycle: 16 h, 20°C/8 h, 16°C) up to the 13-leaf stage. Ryegrass (*Lolium perenne* var. Delika) seeds were sown in perlite and were then transferred to a hydroponic system with nutrient solution (1 mM K_2_SO_4_, 1 mM NH_4_NO_3_, 0.4 mM KH_2_PO_4_, 0.15 mM K_2_HPO_4_, 3 mM CaCl_2_, 0.5 mM MgSO_4_, 0.2 mM Fe-Na EDTA, 14 μM H_3_BO_3_, 5 μM MnSO_4_, 3 μM ZnSO_4_, 0.7 μM (NH_4_)_6_Mo_7_O_24_, 0.7 μM CuSO_4_, 0.1 μM CoCl_2_) in a greenhouse (day/night cycle: 16 h, 20°C/8 h, 16°C) until the development of 4 tillers. All media were sterilized, but plants were not grown under sterile conditions. Plants were thus cultivated in the vegetative phase for approximately 5–6 weeks (before flowering). Several root exudate collection campaigns (numbered I to VII) were performed for the three plant species, each consisting of at least four biological replicates (numbered 1 to 6; Supplementary Table S1). One replicate corresponds to the root exudates of two plants.

### 2.3 Root exudate collection

Plant roots were rinsed briefly with sterile ultrapure water to remove the nutrient solution and then immersed in 500 mL of sterile ultrapure water for 1 h. For rapeseed and ryegrass, one plant was immersed in 500 mL, while for pea, two plants were immersed in the same volume. Water containing root exudates was collected in Falcon tubes and frozen at −20°C for storage and transport. The tubes were then thawed and centrifuged at 10,000 rpm for 15 min to remove cells and debris. Supernatants were collected, frozen, and freeze-dried before being resuspended in sterile ultrapure water, thus being 60-fold more concentrated than the exudation water. These concentrated exudates will be further referred to as root exudate samples. Carbon and nitrogen concentrations of each root exudate sample were determined by IRMS using the PLATIN’ platform (University of Caen Normandy; Supplementary Table S1).

### 2.4 Chemotaxis capillary assay

Capillary assays were adapted from Allard-Massicotte et al. (2016). Bacterial strains were grown on LB to an optical density at 580 nm (OD_580_) of approximately 1 (bacterial density N ≈ 6.10^8^ CFU/mL) and were then washed in chemotaxis buffer (10 mM potassium phosphate buffer pH 7.0, 0.1 mM EDTA, 0.05% glycerol, 5 mM sodium-D,L-lactate, 0.14 mM CaCl_2_, 0.3 mM (NH_4_)_2_SO_4_) for *B. subtilis* or in phosphate-buffered saline (PBS, Gibco™, pH 7.4) for *P. fluorescens* and *A. brasilense*. The OD_580_ of the bacterial suspension was adjusted with buffer to 0.04 for *B. subtilis* (N ≈ 2.10^7^ CFU/mL) and 0.02 for *P. fluorescens* and *A. brasilense* (N ≈ 1.10^7^ CFU/mL). A total of 200 μL of the bacterial suspension was added to each well of a 96-well plate. Microcapillaries were filled with root exudates or with positive (10 mM L-alanine) or negative (buffer) control solutions in triplicate for each condition. The microcapillaries were then dipped in the bacterial suspensions for 45 min at 30°C for *B. subtilis* and *P. fluorescens* or 25°C for *A. brasilense*. After rinsing, the content of the capillary was emptied into a buffer and diluted in order to determine the number of bacteria that had migrated into the capillary by CFU counting on LB agar plates. The ratio of the mean number of bacteria in the exudate capillary to the mean number of bacteria in the negative control capillary was calculated for each experiment. We considered this ratio to indicate a positive chemotactic response if it was ≥3, and a negative response if it was <3. For each root exudate sample, the experiment was replicated at least two times, and the majority response was retained. Then, the minimum attractive concentration (MAC) was determined (see 3.2.1).

### 2.5 Bacterial growth monitoring

Bacterial strains were initially grown in 500 μL M9 medium in hemolysis tubes for 24–32 h. Then, 1 μL of this pre-culture (N ≈ 1.10^9^ CFU/mL) was used to inoculate new tubes containing 250 μL PBS 2X (16 g/L NaCl, 0.4 g/L KCl, 2.88 g/L Na_2_HPO_4_, 0.49 g/L KH_2_PO_4_, pH 7.4) supplemented with 250 μL of root exudates, 500 μL M9 (positive control), or 500 μL PBS only (negative control). Tubes were placed horizontally in the incubator to maximize agitation (120–140 rpm). Bacterial cultures were monitored for 24–40 h with regular enumeration on LB agar plates to calculate bacterial density (N) over time. The growth curves obtained were used to determine the generation time (G) and the bacteria production for each condition. Bacteria production was calculated as the ratio of the bacterial density produced (ΔN = N_max_–N_0_) to the carbon concentration of the root exudate sample or medium ([C]), thus reflecting the number of bacteria produced per gram of carbon present in the medium (ΔN/[C] in CFU/g of carbon). This ratio allows the normalization of media and root exudate concentrations for comparison.

### 2.6 Statistical analyses

Statistical analyses were performed with RStudio software (v4.3.1; R Core Team, 2023). Non-parametric tests were chosen due to the few number of replicates. Comparisons between two conditions were performed using a Wilcoxon–Mann–Whitney test, while comparisons between more than two conditions were performed using a Kruskal–Wallis test, followed by a Dunn *post-hoc* test with Bonferroni correction. Statistical significance was determined when the *p*-value or *p*-adjust was <0.05.

### 2.7 Love match score determination

To compare the bacterial responses to root exudates between plant and bacterial species, each physiological response of each plant–PGPR pair was assigned a score ranging from 0 to 4. This score reflects the effectiveness of the bacterial response (4: excellent response, 3: good response, 2: medium response, 1: poor response, 0: no response). For chemotaxis, 0 indicates no attraction; 1 is assigned if the median MAC ≥ 100 mg C/L; 2 if 100 < MAC ≤ 30 mg C/L; 3 if 30 < MAC ≤ 10 mg C/L; 4 if MAC < 10 mg C/L. When the variability is considerable (MACs ranging between more than 2×log_10_), the score is reduced by 1. For generation time, 0 indicates no growth; 1 is assigned if the mean G on root exudates is significantly higher than a reference medium; 2 if G is higher than M9 medium; 3 if G is similar to or lower than M9 medium; 4 if G is similar to or lower than LB medium. For bacteria production, 0 indicates no growth; 1 is assigned if the mean bacteria production on root exudates is significantly lower than a reference medium; 2 if it is similar to or lower than reference media; 3 if it is higher than reference media; 4 if it is significantly higher than a reference medium. For each plant–PGPR couple, a “love match” score was defined as the sum of these three physiological response scores. The love match score therefore reflects the compatibility of the plant–bacteria pair for rhizocompetence traits.

## 3 Results

### 3.1 Collection and quantification of root exudates

Rapeseed (*Brassica napus*), pea (*Pisum sativum*), and ryegrass (*Lolium perenne*) were cultivated in a phytotronic chamber or a greenhouse under hydroponic conditions, and their root exudates were collected as described in *Materials and methods*. Several collection campaigns were conducted, each providing at least four biological replicates to reflect the natural variability of samples collected within and between campaigns from the same plant (Supplementary Table S1). Indeed, this intra-species variability can affect bacterial responses and contribute to the difficulty of identifying statistically significant differences between plants.

Another technical limitation is the small amount of root exudates harvested. In fact, it was not possible to measure their dry weight. However, using the IRMS analyses, which allow isotopes to be quantified in a small volume (100 μL), we were able to determine the total carbon (C) and nitrogen (N) concentrations of each sample. In this study, we chose to normalize root exudate amounts with their C concentrations, assuming that they reflect the amount of organic molecules contained in the exudates. This allows us to compare bacterial responses between samples and plant species. In fact, it seems to be a reliable indicator for quantifying root exudates, as they are known to represent an important part of soil organic carbon (Dhungana et al., 2023; Lei et al., 2023).

**Figure 1:**
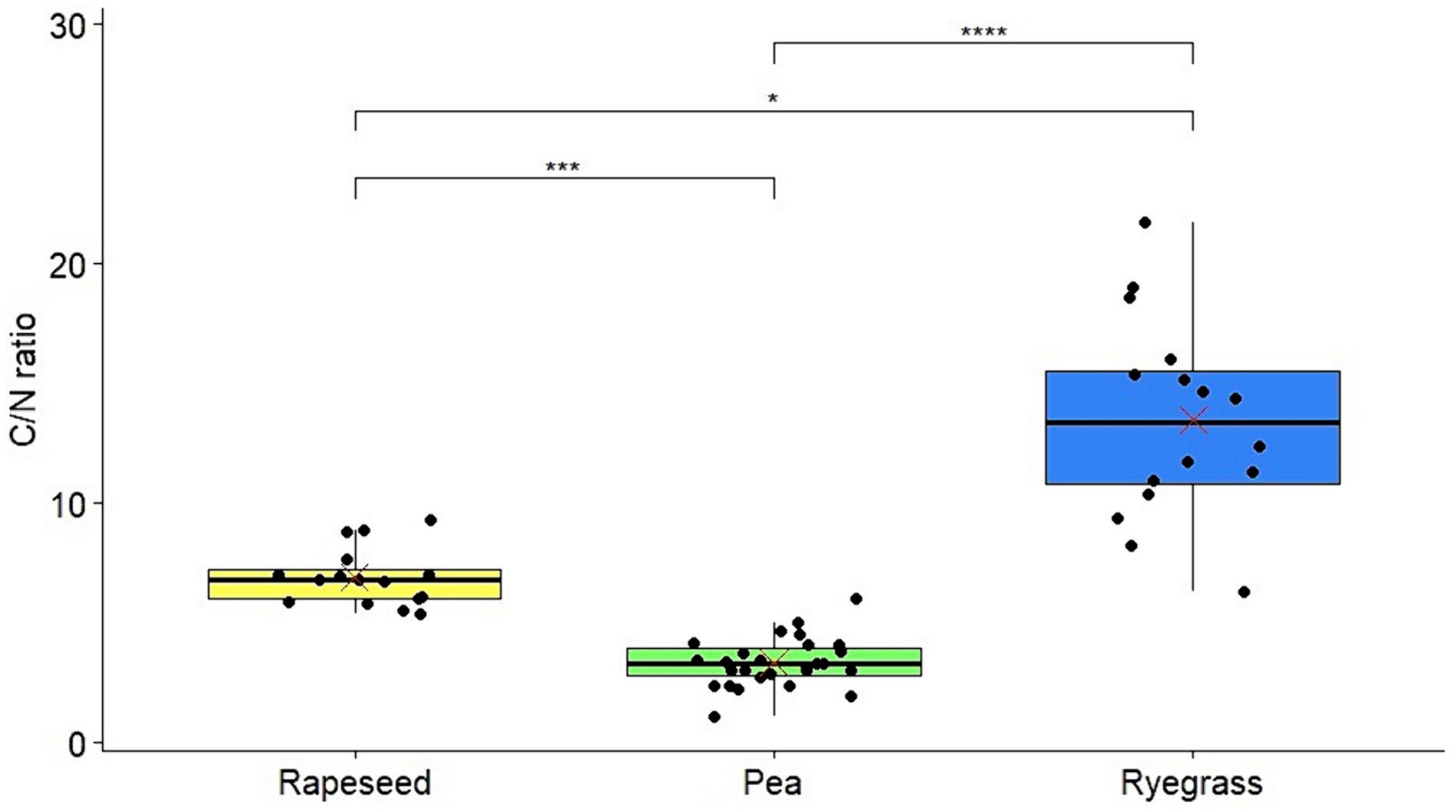
C/N ratios of all root exudate samples from rapeseed, pea, and ryegrass. Red crosses: means; *p-value ≤0.05; ***p-value ≤0.001; ****p-value ≤0.0001.

The C and N concentrations of each sample showed differences between root exudates from the three plant species (Supplementary Table S1). In fact, root exudates had a mean C concentration of 590 mg/L for rapeseed, 48 mg/L for pea, and 370 mg/L for ryegrass, while the mean N concentration was 87 mg/L for rapeseed, 16 mg/L for pea, and 28 mg/L for ryegrass. In addition, C/N ratios were calculated for each of the samples and showed significant differences among the three plant species, with a low ratio for pea root exudates (3.3), an intermediate ratio for rapeseed exudates (6.9), and a high ratio for ryegrass exudates (13.4), with the highest variability (Figure 1). In this study, four to six root exudate samples were used for growth and chemotaxis assays, and their C and N concentrations as well as C/N ratios are presented in Table 1.

**Table 1:** Carbon and nitrogen concentrations and C/N ratios of root exudate samples from rapeseed, pea, and ryegrass used to study bacterial responses.

Plant	RE sample	[C] mg/L	[N] mg/L	C/N ratio
Rapeseed	I-1^b^	270	40	6.8
	I-4^b^	300	50	6.0
	II-1^a^	840	120	7.0
	II-2^a^	910	130	7.0
	II-3^b^	1,150	170	6.8
	II-4^b^	320	60	5.3
	III-1^a^	679	77	8.8
	III-2^a^	571	65	8.8
	III-3^b^	380	41	9.3
	IV-1^a^	721	108	6.7
	IV-2^a^	880	153	5.8
	IV-3^b^	691	119	5.8
Pea	I-1^a^	60	20	3.0
	I-2^a^	58	17	3.4
	II-1^a^	61	15	4.1
	II-2^a^	60	20	3.0
	III-1^b^	112	25	4.5
	III-2^a^	85	21	4.0
	III-3^a^	51	11	4.6
	V-1^b^	33	15	2.2
	VI-1^b^	49	13	3.8
	VII-1^b^	30	9	3.3
Ryegrass	I-1^a^	320	20	16.0
	I-3^b^	380	20	19.0
	I-4^b^	310	30	10.3
	II-1^a^	292	25	11.7
	II-2^a^	339	31	10.9
	II-3^b^	361	32	11.3
	III-1^a^	363	24	15.1
	III-2^a^	137	22	6.2
	III-3^b^	539	29	18.6
	IV-1^a^	585	27	21.7
	IV-4^b^	482	39	12.4

RE, Root Exudate sample from collection campaign no I-VII and biological replicate no 1, 2, 3, 4; [C], Carbon concentration; [N], Nitrogen concentration.

a sample used for chemotaxis assays.

b sample used for growth assays.

### 3.2 Chemotaxis responses to root exudates

#### 3.2.1 Definition of the minimum attractive concentration

The chemotactic responses of *Bacillus subtilis* ATCC 6633, *Pseudomonas fluorescens* ATCC 17400, and *Azospirillum brasilense* Sp245 to root exudates from rapeseed, pea, and ryegrass were studied. For each plant, six root exudate samples from at least three different collection campaigns were tested to account for their biological variability (Table 1, samples^a^). In order to quantify bacterial chemotaxis in response to root exudates, we defined a new indicator, the minimum attractive concentration (MAC), which corresponds to the lowest exudate concentration tested capable of attracting a given bacterium. Exudate MAC was determined by serial decimal dilutions of each sample, followed by a chemotaxis assay for each dilution until the chemotactic response was extinguished (Table 2). Thus, the MACs reflect both the attractiveness of the exudates and the sensitivity of the bacteria to them. Because MACs were determined from a set of discontinuous values separated by a factor of 10, their medians were examined rather than their means.

**Table 2:** Chemotactic responses of *Bacillus subtilis (Bs)*, *Pseudomonas fluorescens (Pf)*, and *Azospirillum brasilense (Ab)* to root exudates from rapeseed, pea, and ryegrass.

Rapeseed					Pea					Ryegrass				
RE	[C] mg/L	*Bs*	*Pf*	*Ab*	RE	[C] mg/L	*Bs*	*Pf*	*Ab*	RE	[C] mg/L	*Bs*	*Pf*	*Ab*
II-1	840	+	+	+	I-1	30*	+	+	+	I-1	320	−	+	+
	84	+	+	nd		6	−	−	+		32	−	+	nd
	8.4	+	+	+		0.6			+		3.2		−	+
	0.8	+	−	+		0.06			−		0.3			−
	0.08	−		−										
II-2	910	+	+	+	I-2	58	+	+	+	II-1	292	−	+	+
	91	+	+	nd		5.8	+	+	+		29.2	−	−	nd
	9.1	+	+	+		0.6	−	−	−		2.9			nd
	0.9	+	−	+							0.2			+
	0.09	−		−							0.02			−
III-1	679	+	+	+	II-1	61	+	+	+	+	339	+	+	+
	67.9	+	+	nd		6.1	−	+	−		33.9	+	+	nd
	6.8	+	+	+		0.6		−			3.4	−	−	+
	0.7	−	−	+							0.3			−
	0.07			−										
III-2	571	+	+	+	II-2	60	+	+	+	III-1	363	−	+	+
	57.1	+	+	nd		6	−	−	+		36.3	−	+	nd
	5.7	+	+	+		0.6			+		3.6		−	nd
	0.6	−	−	−		0.06			−		0.4			+
											0.04			−
IV-1	721	+	+	+	III-2	85	+	+	+	III-2	137	+	+	+
	72.1	+	+	nd		8.5	−	+	+		13.7	+	+	nd
	7.2	+	+	+		0.9		−	+		1.4	−	−	+
	0.7	−	−	−		0.09			+		0.1			−
						0.009			−					
IV-2	880	+	+	+	III-3	51	+	+	+	IV-1	585	−	+	+
	88	+	+	nd		5.1	−	−	−		58.5	−	+	nd
	8.8	+	−	+							5.9		−	+
	0.9	−		−							0.6			−

RE, Root Exudate sample from collection campaign no I, II, III, IV and biological replicate no 1, 2, 3; [C], Carbon concentration; nd, not determined; *Sample I-1 of pea root exudates was initially concentrated 30-fold instead of 60-fold like all the other samples. Gray boxes correspond to the Minimum Attractive Concentration.

#### 3.2.2 Root exudates differentially attract beneficial bacteria

**Figure 2:**
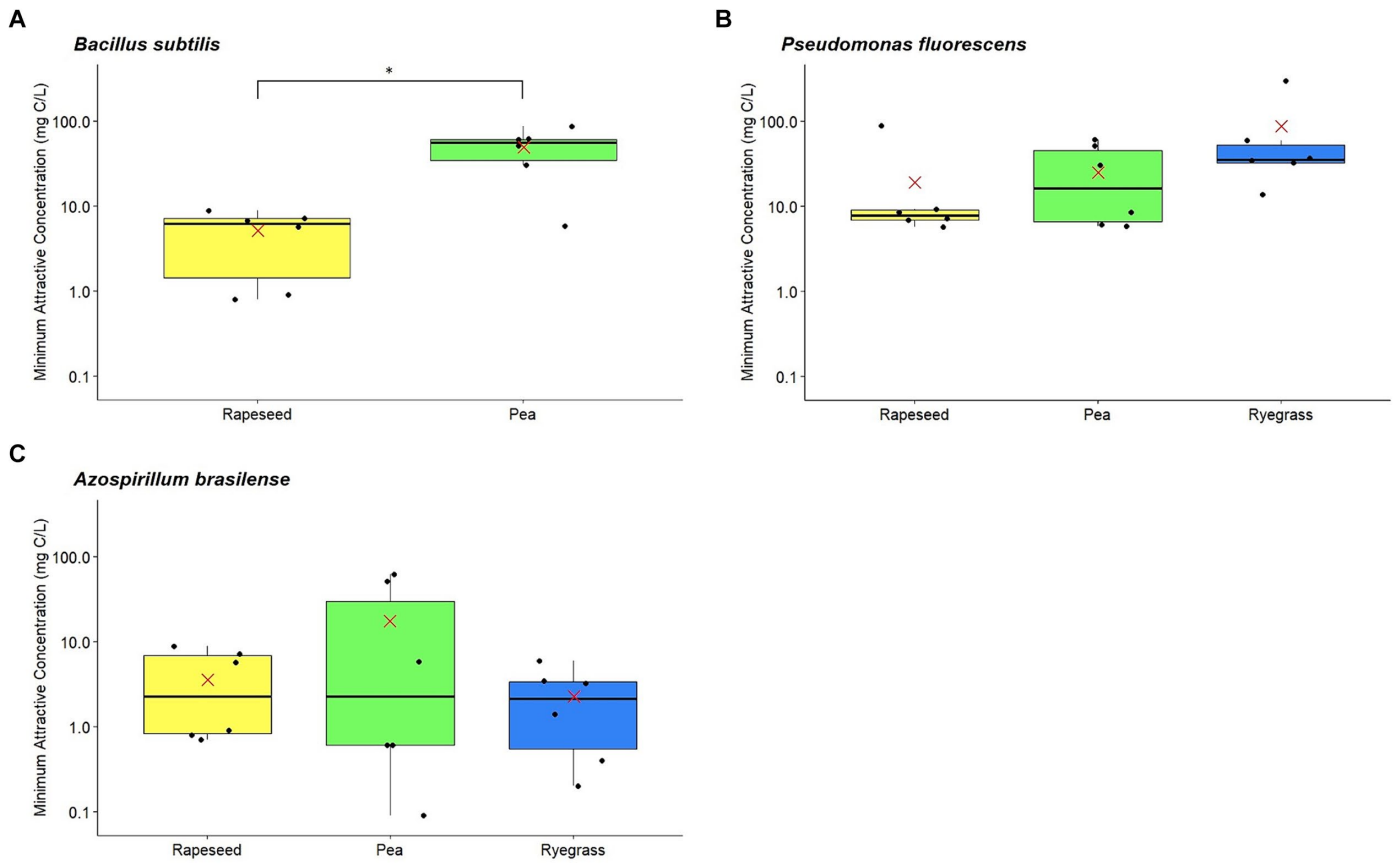
Minimum Attractive Concentrations (MACs) of *Bacillus subtilis* **(A)**, *Pseudomonas fluorescens* **(B)**, and *Azospirillum brasilense* **(C)** in response to root exudates from rapeseed, pea, and ryegrass (log scale). Red crosses: means; *p-value ≤0.05.

*B. subtilis* ATCC 6633 presented a positive chemotactic response to all root exudate samples from rapeseed and pea. In contrast, four of the six ryegrass exudate samples did not induce any chemotactic response, whether concentrated or 10-fold diluted, making it impossible to determine their MACs (Table 2). Thus, we excluded ryegrass root exudates for statistical analyses because we considered that they were either of low attractiveness or that *B. subtilis* ATCC 6633 had little to no sensitivity to them. When comparing rapeseed and pea MACs, rapeseed root exudates had a significantly lower MAC (median = 6.25 mg C/L) than pea exudates (median = 55.5 mg C/L; Figure 2A). Together, these results show that *B. subtilis* ATCC 6633 is able to distinguish between different exudates, and is preferentially attracted to rapeseed exudates, then to pea exudates, and has little or no attraction to ryegrass exudates.

*P. fluorescens* ATCC 17400 presented a positive chemotactic response to all root exudate samples from the three plants tested. When the MACs of each plant species were determined and compared, no significant difference was observed, probably due to the high variability of MACs within root exudate samples from the same plant. Nevertheless, trends can be observed between the three plants, with increasing median MACs between rapeseed (median = 7.8 mg C/L), pea (median = 19.2 mg C/L), and ryegrass (median = 35.1 mg C/L; Figure 2B). These observations suggest that *P. fluorescens* ATCC 17400 may be preferentially attracted to root exudates following the same pattern as *B. subtilis* ATCC 6633.

*A. brasilense* Sp245 also presented a positive chemotactic response to all root exudate samples from the three plants tested. No significant difference was observed between the three plant MACs. Moreover, *A. brasilense* Sp245 response to root exudates was more variable between samples from pea than from the two other plants, with MACs ranging from 0.09 to 61 mg C/L (Figure 2C). Interestingly, unlike the other two PGPR, no trend appears to be visible between the median MACs of root exudates from rapeseed (median = 3.3 mg C/L), pea (median = 3.2 mg C/L), and ryegrass (median = 2.3 mg C/L). This suggests that *A. brasilense* Sp245 is attracted with the same high efficiency (low MACs) by all tested root exudates regardless of the plant species from which they are obtained.

The chemotaxis results show that *B. subtilis* ATCC 6633 is preferentially attracted to rapeseed, then pea, and finally ryegrass root exudates, and that *P. fluorescens* ATCC 17400 may have similar preferences, while *A. brasilense* Sp245 appears to be equally attracted to root exudates from all three plants, although the variability between samples is considerable for pea exudates.

### 3.3 Bacterial growth on root exudates

#### 3.3.1 Growth parameter determination

In order to assess the ability of PGPR to catabolize and utilize root exudates to produce biomass, it was necessary to develop a bacterial growth monitoring system adapted to small cultivation volumes due to the limited amount of harvested exudates. Therefore, bacteria were cultivated in 500 μL of medium in hemolysis tubes. Growth on a rich medium (LB) and a minimal medium (M9) were studied as controls. For each plant, four root exudate samples from four different collection campaigns were used for growth assays to reflect their biological variability (Table 1, samples^b^). To avoid osmotic shock and/or pH fluctuations during culture, root exudate samples were supplemented with PBS. Thus, the availability of essential macroelements in sufficient amounts for bacterial growth was tested.

Interestingly, we observed that *B. subtilis* ATCC 6633, *P. fluorescens* ATCC 17400, and *A. brasilense* Sp245 could grow on all root exudate samples from rapeseed, pea, and ryegrass, without other nutrients. This shows that root exudates can provide all the macroelements (C, N, S, P, K, Ca, Mg, and Fe) that are necessary for bacterial growth in a bioavailable form. To analyze bacterial growth, generation times and bacteria production were determined. The bacteria production was expressed as the number of bacteria produced per gram of carbon in the root exudate sample or reference medium. Reference media had a C concentration of 6.62 g/L for LB, 1.59 g/L for the M9 used for *B. subtilis* and *P. fluorescens*, and 1.04 g/L for the M9 used for *A. brasilense*.

#### 3.3.2 Beneficial bacteria have different growth parameters depending on root exudates

**Figure 3:**
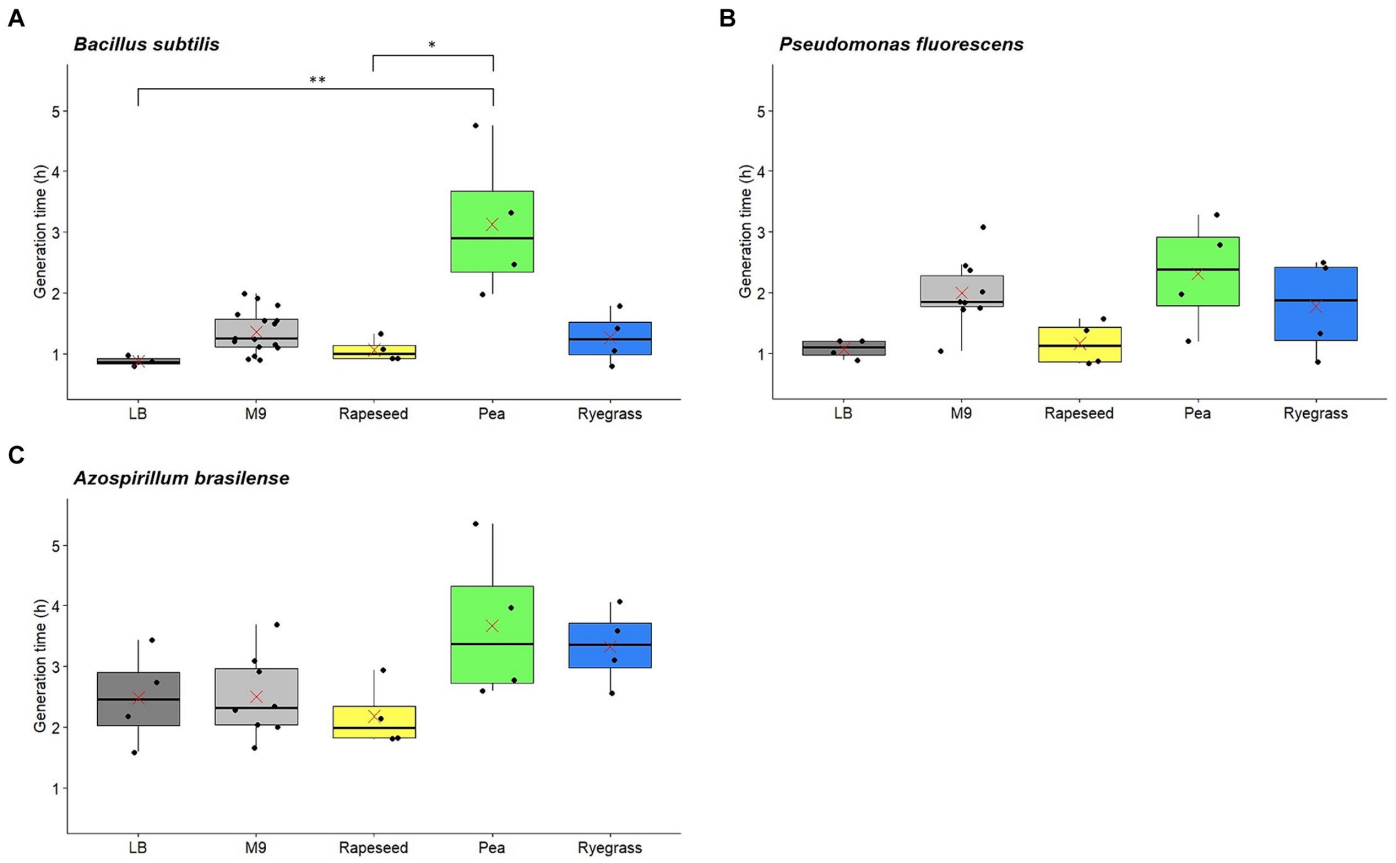
Generation times of *Bacillus subtilis* **(A)**, *Pseudomonas fluorescens* **(B)**, and *Azospirillum brasilense* **(C)** during growth on rich (LB) or minimal (M9) media as well as on root exudates from rapeseed, pea, and ryegrass. Red crosses: means; *p-value ≤0.05; **p-value ≤0.01.

**Figure 4:**
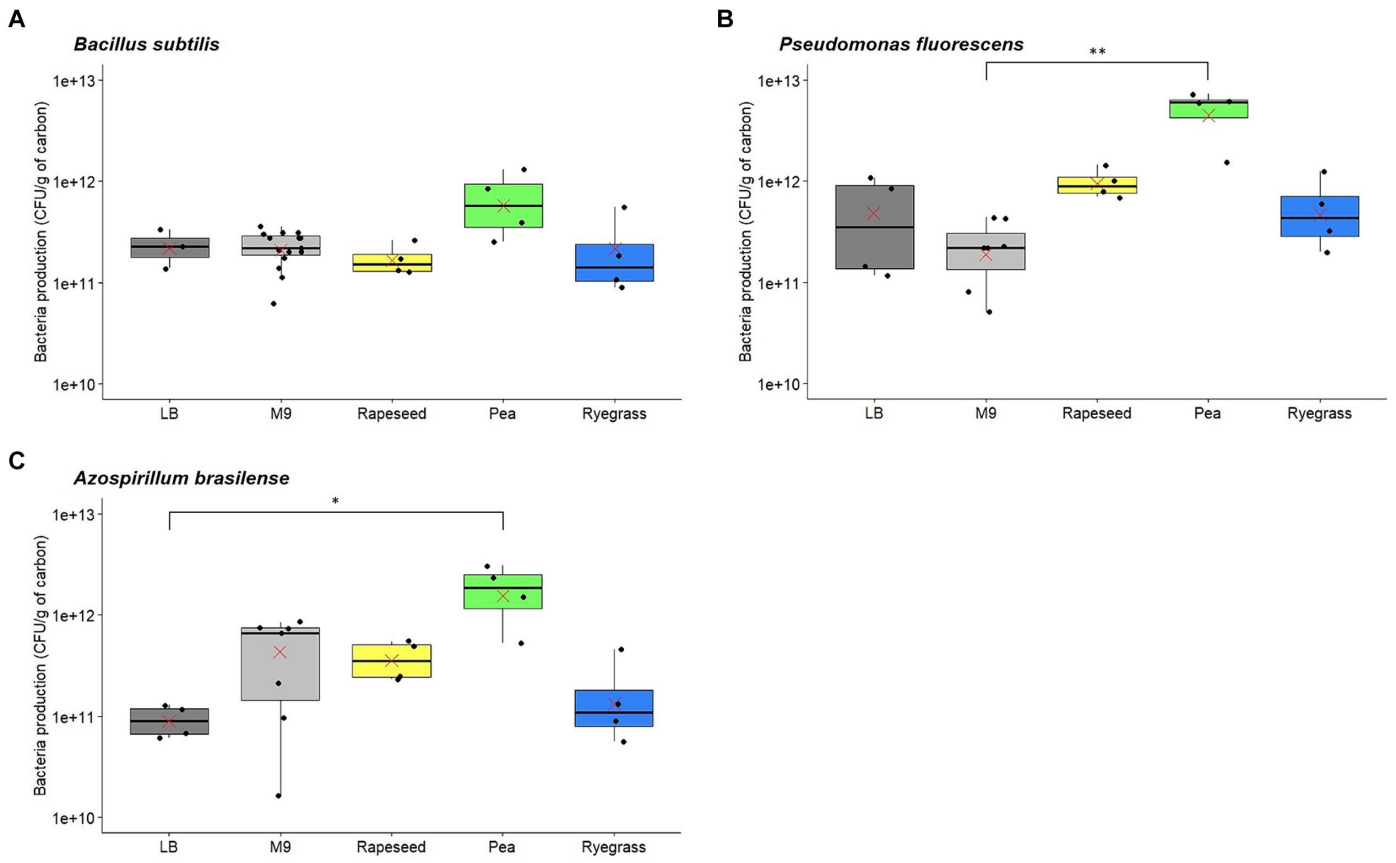
Bacteria production of *Bacillus subtilis* **(A)**, *Pseudomonas fluorescens* **(B)**, and *Azospirillum brasilense* **(C)** after growth on rich (LB) or minimal (M9) media as well as on root exudates from rapeseed, pea, and ryegrass (log scale). Red crosses: means; *p-value ≤0.05; **p-value ≤0.01.

For *B. subtilis* ATCC 6633, the mean generation time on the M9 medium (G = 1.36 h) appeared longer than on the LB medium (G = 0.88 h), consistent with their status as minimal and rich media, respectively. The generation time on pea root exudates (G = 3.13 h) was significantly longer than on LB medium and on rapeseed exudates (G = 1.06 h), which had the shortest generation times (Figure 3A). Regarding ryegrass exudates, an intermediate generation time was observed (G = 1.26 h). The mean bacteria productions of *B. subtilis* ATCC 6633 obtained for LB (2.32 × 10^11^ CFU/g of carbon) and M9 (2.29 × 10^11^ CFU/g of carbon) media, as well as for rapeseed (1.73 × 10^11^ CFU/g of carbon) and ryegrass (2.33 × 10^11^ CFU/g of carbon) root exudates were similar (Figure 4A). In contrast, bacteria production tended to be higher for pea exudates (6.99 × 10^11^ CFU/g of carbon), although no significant difference was observed. Thus, while pea root exudates induced the longest generation time, they also appeared to allow the highest bacteria production.

For *P. fluorescens* ATCC 17400, the mean generation time on the M9 medium (G = 1.99 h) appeared longer than on the LB medium (G = 1.07 h), as for *B. subtilis* ATCC 6633. Moreover, trends can be observed between the mean generation times for the three plants, with a short generation time for rapeseed exudates (G = 1.16 h), followed by an intermediate one for ryegrass exudates (G = 1.77 h), and finally, a longer generation time for pea exudates (G = 2.31 h; Figure 3B). Yet, no statistically significant difference was observed, which may be explained by the variability between replicates. *P. fluorescens* ATCC 17400 had similar mean bacteria productions after its growth on LB medium (5.49 × 10^11^ CFU/g of carbon), M9 medium (2.37 × 10^11^ CFU/g of carbon), and ryegrass exudates (5.89 × 10^11^ CFU/g of carbon; Figure 4B). However, the bacteria production reached on pea root exudates was significantly higher (5.21 × 10^12^ CFU/g of carbon) than on the M9 medium. Although not significant, bacteria production on rapeseed exudates appeared to be slightly higher (9.82 × 10^11^ CFU/g of carbon) than on reference media or ryegrass exudates. These results suggest that *P. fluorescens* ATCC 17400 had different generation times depending on root exudates, following the same pattern as *B. subtilis* ATCC 6633. Pea root exudates also allowed the highest bacteria production for *P. fluorescens* ATCC 17400.

For *A. brasilense* Sp245, there seemed to be no difference between the mean generation times of the M9 medium (G = 2.50 h) and the LB medium (G = 2.48 h). These values are consistent with the results of Carreras et al. (2019). Trends can be observed for root exudates, with a shorter mean generation time when grown on rapeseed exudates (G = 2.18 h) than on pea (G = 3.67 h) and ryegrass exudates (G = 3.33 h), although these differences were not significant (Figure 3C). This suggests that rapeseed exudates allowed the fastest growth of *A. brasilense* Sp245, while both pea and ryegrass exudates induced a longer generation time. The mean bacteria productions of *A. brasilense* Sp245 obtained on LB (9.33 × 10^10^ CFU/g of carbon) and M9 (4.76 × 10^11^ CFU/g of carbon) media, as well as on rapeseed (3.80 × 10^11^ CFU/g of carbon) and ryegrass (1.84 × 10^11^ CFU/g of carbon) root exudates were relatively comparable (Figure 4C). In contrast, bacteria production after growth on pea root exudates was significantly higher (1.86 × 10^12^ CFU/g of carbon) than on LB medium.

The growth results suggest that the generation times of *B. subtilis* ATCC 6633, *P. fluorescens* ATCC 17400, and *A. brasilense* Sp245 are shorter on rapeseed than ryegrass, and longer on pea root exudates. For all three PGPR, pea root exudates allowed a greater bacteria production than reference media or exudates from the other two plants, although this trend was not found to be significant at the *p* < 0.05 confidence level in all the comparisons.

### 3.4 Love match score compares the compatibility of plant–bacteria pairs

To evaluate, compare, and rank the compatibility of each plant–PGPR pair, each physiological response of *B. subtilis* ATCC 6633, *P. fluorescens* ATCC 17400, and *A. brasilense* Sp245 to root exudates from rapeseed, pea, and ryegrass was assigned an efficiency score ranging from 0 to 4 (Table 3; see 2.7). For each plant–PGPR couple, the sum of these scores was calculated and named the love match score, highlighting the best combinations between plants and bacteria.

**Table 3:** Love match scores between *Bacillus subtilis*, *Pseudomonas fluorescens*, *Azospirillum brasilense*, and root exudates from rapeseed, pea, or ryegrass based on chemotactic responses, generation times, and biomass production.

		Rapeseed	Pea	Ryegrass
*Bacillus subtilis*	Chemotactic response	4	2	0
	Generation time	4	1	3
	Biomass production	2	3	2
	**Love match**	**10**	**6**	**5**
*Pseudomonas fluorescens*	Chemotactic response	4	3	2
	Generation time	4	2	3
	Biomass production	3	4	2
	**Love match**	**11**	**9**	**7**
*Azospirillum brasilense*	Chemotactic response	4	3	4
	Generation time	4	2	2
	Biomass production	3	4	2
	**Love match**	**11**	**9**	**8**

Each bacterial response was assigned a score from 0 to 4 according to its effectiveness. 4: excellent response, 3: good response, 2: medium response, 1: poor response, 0: no response. The love match score of each plant–bacteria couple is calculated as the sum of these three scores. Bold values: love match score (see 2.7).

When focusing on bacteria, *A. brasilense* Sp245 and *P. fluorescens* ATCC 17400 had the best love match scores, with responses ranging from “medium” to “excellent” for all root exudates. In contrast, *B. subtilis* ATCC 6633 had lower love match scores, especially for pea and ryegrass exudates, for which the poorest generation time and chemotaxis were observed, respectively. When comparing root exudates from the three plant species, rapeseed exudates had the best efficiency, with love match scores ranging from 10 to 11, as they were highly attractive and allowed rapid bacterial growth for all PGPR. Pea exudates had intermediate love match scores ranging from 6 to 9, with varying efficiencies depending on the bacterial behavior studied. In particular, pea exudates induced slow bacterial growth but high biomass production. Finally, ryegrass exudates had the lowest love match scores ranging from 5 to 8, for which *B. subtilis* ATCC 6633, *P. fluorescens* ATCC 17400, and *A. brasilense* Sp245 showed various responses.

## 4 Discussion

Root exudates include a high number of complex and diverse compounds that are released into the soil and select rhizosphere microorganisms, thus enabling the establishment of the rhizosphere microbiota (Sasse et al., 2018; Ma et al., 2022). The composition of root exudates is dynamic as it is modulated by each plant depending both on its genotype as well as its biotic and abiotic environment, which consequently modulates the rhizosphere microbial communities by selecting specific microorganisms (Sharma et al., 2023). However, there is currently no exhaustive list of the many components of a plant’s root exudates, and the precise mechanisms by which exudates select microorganisms are not well understood. In this study, we aimed to identify specific bacterial responses depending on the plant species. Therefore, the chemotaxis and growth of three PGPR (*Bacillus subtilis* ATCC 6633, *Pseudomonas fluorescens* ATCC 17400, and *Azospirillum brasilense* Sp245) were evaluated in response to root exudates harvested from rapeseed, pea, and ryegrass.

First, chemotaxis to root exudates was studied, since it is the first behavior of beneficial bacteria involved in the rhizosphere microbiota assembly (Feng et al., 2021). Regarding plant species, these results show that root exudates from rapeseed, pea, and ryegrass differentially attract beneficial bacteria depending on the plant species, which is consistent with their apparent diverse composition among genotypes (Herz et al., 2018; Vives-Peris et al., 2020). This plant-specific composition of root exudates could also be involved in rhizosphere signaling and may explain how plants shape their root microbiota and select specific microorganisms, especially PGPR (Jacoby et al., 2020; Upadhyay et al., 2022). Indeed, Vora et al. (2021) showed that root exudates from pigeon pea and maize, mono or co-cultivated, differentially attracted the PGPR strains *Enterobacter* sp. C1D, *Pseudomonas* sp. G22, and *Rhizobium* sp. IC3109, resulting in specific bacterial root colonization. Interestingly, we observed that rapeseed root exudates were highly attractive to all three PGPR, indicating the presence of abundant and/or strong chemoattractants. These could include specific organic acids such as malate, citrate, and fumarate, and sugars like fructose which have been identified in rapeseed root exudates and also described as chemoattractants for the PGPR strain *Bacillus amyloliquefaciens* SQR9 (Feng et al., 2018; Delamare et al., 2023).

Regarding bacterial species, our results indicate that *B. subtilis* ATCC 6633, *P. fluorescens* ATCC 17400, and *A. brasilense* Sp245 are attracted with different efficiencies to root exudates from the same plant. In particular, *A. brasilense* is similarly attracted to root exudates from all three plants, which is consistent with a recent study reporting that this PGPR is attracted to all exudate compounds from pea, tomato, and cucumber (Nisha et al., 2024). In addition, bacterial responses to ryegrass exudates range from a high level of attraction for *A. brasilense* Sp245 to no attraction at all for *B. subtilis* ATCC 6633. This could be explained by chemoattractants which only have an effect on *A. brasilense* Sp245 and *P. fluorescens* ATCC 17400, or by specific repulsive compounds in ryegrass exudates that are only perceived by *B. subtilis* ATCC 6633. Indeed, Feng et al. (2018) reported that although the PGPR strain *B. amyloliquefaciens* SQR9 had a general positive chemotactic response to cucumber root exudates, some of their components, such as salicylic acid, were characterized as repellents. Therefore, we can hypothesize that such repellent compounds may be present in some or all of the ryegrass root exudate samples, but at varying concentrations among them.

Hence, the plant attracts specific microorganisms and promotes their rhizosphere colonization by releasing a particular root exudate profile (Allard-Massicotte et al., 2016; Liu et al., 2020; Saleh et al., 2020). To do so, it implies that bacteria are able to catabolize at least some of the molecules present in root exudates and use them as nutrients for growth. However, only a few studies have demonstrated this. Guyonnet et al. (2018) showed that plants can stimulate microbial activities through root exudation and modify the diversity of microorganisms involved in root exudate assimilation. Zhalnina et al. (2018) studied the substrate preferences of soil bacterial isolates growing on *Avena barbata* root exudates and identified an increased uptake of certain compounds, such as aromatic organic acids, by rhizosphere bacteria. Moreover, Carreras et al. (2019) showed that *A. brasilense* was able to utilize root exudates from three Sahelian woody species as carbon and nitrogen sources with different growth parameters (G and bacteria production) among species, indicating a preference for the most drought-tolerant of the three plant species. Recently, Dhungana et al. (2023) observed that rhizosphere bacterial isolates from three distinct plant species grew differently in their root exudates, and not necessarily better in their host’s exudates, but rather depending on the plant species.

To provide new insights on the particular differences induced by root exudates on the growth behavior of beneficial bacteria, *B. subtilis* ATCC 6633, *P. fluorescens* ATCC 17400, and *A. brasilense* Sp245 were cultivated in rapeseed, pea, and ryegrass exudates and monitored by CFU counting. For all three PGPR, the fastest growth was obtained with rapeseed exudates, suggesting that they contain a rich amount of nutrients that are easy and fast to catabolize. These effective nutrients may be different for the three PGPR because they have different metabolisms. In contrast, slower growth was obtained with pea exudates, suggesting that they contain complex nutrients that are more difficult to catabolize and/or antimicrobial molecules. Indeed, pea root exudates are directly involved in plant protection against biotic stresses through proteins such as chitinases, glucanases, or arabinogalactan proteins, as well as phenolic compounds such as stilbenes, which are antimicrobial molecules (Makarova et al., 2007; Wen et al., 2007; Fortier et al., 2023). Moreover, legumes like pea are capable of producing specific metabolites such as soyasaponins, which can act as antibacterial agents (Seitz et al., 2023). Although rapeseed root exudates can also contain antimicrobials (Fang et al., 2016), pea exudates may therefore considerably affect bacterial growth through these compounds.

To complete growth analyses, the bacteria production was determined and expressed as the number of bacteria produced per gram of carbon contained in the culture medium. For the same amount of carbon, pea root exudates allowed a higher bacteria production for *B. subtilis* ATCC 6633, *P. fluorescens* ATCC 17400, and *A. brasilense* Sp245 compared to reference media and other exudates. This could be explained by the ability of the PGPR studied to efficiently transform the nutrients present in pea exudates into bacterial biomass and/or by the catabolism of low-carbon molecules that could be nitrogen-rich instead. In fact, we observed that pea exudates had the lowest C/N ratio of all root exudates, indicating an important release of nitrogen by pea roots. This is consistent with the literature describing high nitrogen rhizodeposition by legumes such as pea, through the exudation of ammonium, amino acids, or ureides, among others (Schmidtke, 2005; Wichern et al., 2008; Fustec et al., 2010).

These growth studies were delicate due to the limited amount of root exudates collected, but we were able to show that root exudates from three different plant species provided the three PGPR studied with all the macroelements required for cell growth. However, bacteria effectively discriminate between these complex molecular cocktails, as the rate and efficiency with which they utilize nutrients differ between plant species. Antimicrobial compounds can also interfere with their growth. Thus, the different composition of root exudates from rapeseed, pea, and ryegrass has a direct and specific effect on the growth of PGPR, and consequently on their long-term establishment in the rhizosphere.

To compare PGPR responses to root exudates between plant and bacterial species, the love match score was defined and calculated for each plant–bacteria pair to reflect their compatibility for the three rhizocompetence traits studied. Interestingly, *P. fluorescens* ATCC 17400 and *A. brasilense* Sp245 had higher love match scores than *B. subtilis* ATCC 6633. Although all three bacteria are well-known PGPR (Basu et al., 2021), it is important to mention that *A. brasilense* and *P. fluorescens* are Gram-negative bacteria, while *B. subtilis* is a Gram-positive bacterium, which implies that they have different physiology and metabolism that could affect their response to root exudates. For plant species, rapeseed root exudates had the best love match scores. This may be explained by specific exudate components such as glucosinolates, which are characteristic of Brassicaceae species like rapeseed and have been shown to select microbial populations such as PGPR from soil (Bressan et al., 2009; Siebers et al., 2018). Moreover, the rapeseed rhizosphere has been shown to harbor phosphate-solubilizing bacteria with plant growth-promoting properties, thus reflecting the ability of rapeseed to attract and recruit these bacteria (Valetti et al., 2018). In contrast, ryegrass root exudates had the lowest love match scores. Although ryegrass exudates have been reported to recruit beneficial bacteria of the genus *Pseudomonas* under salt stress (Cao et al., 2024), the exact compounds responsible for this selection remain poorly described and may be less effective than those from other plant species.

In conclusion, the need for sustainable agroecosystems in the context of climate change is now undeniable (George et al., 2024). PGPR such as *B. subtilis*, *P. fluorescens,* and *A. brasilense* have been extensively described for their beneficial effects on crop production (Basu et al., 2021; Shah et al., 2021), while root exudates appear to be key to their recruitment and retention (Hassan et al., 2019; Jacoby et al., 2020). However, the particular mechanisms responsible for the specific selection of rhizosphere microorganisms by the plant remain unclear. In this study, we provided new insights into the efficiency with which *B. subtilis* ATCC 6633, *P. fluorescens* ATCC 17400, and *A. brasilense* Sp245 respond to root exudates from rapeseed, pea, and ryegrass, and revealed important differences between the exudates of each plant involved in the first steps of rhizosphere colonization, i.e., chemotaxis and bacterial growth. *P. fluorescens* ATCC 17400 and *A. brasilense* Sp245 seemed to respond more efficiently to root exudates than *B. subtilis* ATCC 6633, while root exudates from rapeseed appeared to be more efficient in these interactions, followed by pea and lastly ryegrass. We also proposed to evaluate each plant–PGPR couple with a love match score, which rates the performance of root exudates in enhancing bacterial rhizocompetence. The love match score represents a new indicator to identify efficient plant–PGPR combinations in the context of bioinoculant use and sustainable agriculture. This scoring system may thus be used to compare other plant–bacteria pairs, as well as be further developed and improved by adding more parameters. The love match score could include other rhizocompetence traits such as the ability to form biofilms, which is another important step for bacterial establishment in the microbiota (Santoyo et al., 2021).

In light of this study, further research will also help decipher the molecular mechanisms involved in the different physiological responses of bacteria, along with which particular compounds or molecular associations are responsible for this specificity, using transcriptomic and metabolomic studies such as mass spectrometry (Escolà Casas and Matamoros, 2021; Calabrese et al., 2023). Field studies will also allow us to validate our findings under realistic conditions (Manfredini et al., 2021; Li et al., 2022). This research will contribute to our understanding of the benefits of root exudates and how they can be exploited to develop rhizosphere engineering (Kumar and Dubey, 2020; Bano et al., 2021; Orozco-Mosqueda et al., 2022). Indeed, manipulating root exudate composition through plant genetic engineering or soaking seeds with exudates has been shown to shape the rhizosphere microbiota and improve colonization by beneficial bacteria such as *A. brasilense* (Barbosa et al., 2020; Kawasaki et al., 2021). Root exudates could thus be used directly or indirectly through intercropping systems as a tool to improve the functions of the rhizosphere microbiota (Preece and Peñuelas, 2020; Li et al., 2023). Therefore, knowing the efficacy of root exudates from a donor plant in attracting and feeding one or more PGPR is a major advantage in selecting which exudates to apply to a recipient plant or in optimizing appropriate plant combinations in intercropping systems in order to promote bioinoculant establishment in the rhizosphere.

## Data Availability

The original contributions presented in the study are included in the article/Supplementary material, further inquiries can be directed to the corresponding author.

## Supplementary material

SUPPLEMENTARY TABLE 1 Carbon and nitrogen concentrations and C/N ratios of all root exudate samples.
